# Analysis of gradual diameter thrombectomy stent *in vitro*

**DOI:** 10.1515/tnsci-2022-0351

**Published:** 2025-01-18

**Authors:** Caifeng Shen, Zhenjian Ma, Hong Li, Ming Wei

**Affiliations:** Clinical College of Neurology, Neurosurgery and Neurorehabilitation, Tianjin Medical University, Tianjin, 300350, China; Department of Surgery, Ecological City Hospital, Tianjin Fifth Central Hospital, Tianjin, 300451, China; Department of Neurosurgery, The Second Hospital of Tianjin Medical University, 23 Pingjiang Road, Tianjin, 300211, China; Department of Neurosurgery, Tianjin Huanhu Hospital, 6 Jizhao Road, Tianjin, 300350, China

**Keywords:** thrombectomy

## Abstract

**Objective:**

To analyze the effect of gradient thrombectomy stent *in vitro*.

**Methods:**

The cerebrovascular fluid circulation model was made and fixed on the test table. About 0.9% sodium chloride injection was injected to the vascular model through a miniature water pump to circulate it. Four kinds of thrombus products containing different red blood cell (RBC) volumes of 0, 5, 40, and 80% were placed at the target location as thrombus. Each type of thrombus was removed five times using SOLITAIRE stent and gradient thrombectomy stent. The thrombectomy effects were observed by comparison of the two types of stents.

**Results:**

Thrombus containing 80% volume of RBC escaped thrombus fragments during thrombectomy with both stents. Thrombus containing 0% volume of RBC (rich in fibrin) had poor chimerism with stents. In the thrombus containing 0% RBC volume, there was no statistically significant difference between the two stents (*p* = 1). Thrombus removal was successful in all cases.

**Conclusion:**

The gradient thrombectomy stent is as effective as the currently used thrombectomy stent *in vitro* studies, and there is much room for improvement.

## 1 Introduction

Stroke is an acute cerebrovascular disease, including ischemic stroke and hemorrhagic stroke, and is the number one killer of adults in China in terms of death and disability. Large vessel occlusions, including the internal carotid artery, main middle artery, basilar artery, and intracranial vertebral artery, account for a high proportion of acute ischemic strokes (AIS). Large vessel occlusions that cannot be successfully recanalized have a poor prognosis of 91–95% and are a class of malignant disease. At present, stent thrombectomy has become the standard treatment method for revascularization of large vessel occlusion. The American Stroke Association guidelines clearly state that mechanical thrombectomy is recommended for eligible acute large vessel occlusion strokes (Class IA recommendation) [1,2]. Endovascular treatment of AIS with thrombectomy stents has achieved high rates of revascularization. At the same time, given the sensitivity of the cerebral vasculature to mechanical injury, the vascular damage produced by the device itself cannot be ignored [3]. Moreover, tortuosity of the occluded vessel is associated with a lower success rate [4,5]. An important issue in the design of stents is how to maximize their effect on restoring cerebral blood flow in a way that minimizes the damage to the vessel.

The common embolization devices currently on the market are of a “straight” design. The intracranial vessels are tortuous, and the intracranial and extra-cranial segments vary greatly in diameter. This prevents the stent from fitting well into the vessel wall during embolization, and the distal vessel is often subjected to greater stress, resulting in greater frictional forces and vessel deformation with a greater potential for vascular injury. This effect may be amplified in curved vessels, thus exacerbating vascular injury. Innovative designs of variable-diameter stents for thrombectomy may minimize damage to the vessel wall and improve the safety of the procedure. This study aimed to perform *in vitro* thrombus removal experiments using different types of synthetic thrombus in an *in vitro* cerebrovascular circulation model [6]. Moreover, we confirmed the effectiveness of the thrombus removal effect and provided a basis for optimizing the design of the graduated diameter stent structure.

## 2 Materials and methods

### 2.1 Materials

Materials used were an *in vitro* model of cerebrovascular circulation using silicone vessels to simulate the intracranial circulatory system. A 37°C saline reservoir and a micro-pump (together called a mini-temperature controlled pump), arterial catheter sheath, black loach guidewire, Navien catheter, and micro-guide wire. A gradual diameter stent with Solitaire stent developed by finite element analysis. Four thrombotic products containing 0, 5, 40, and 80% of red blood cell (RBC) volume and 0.9% sodium chloride injection. The new stent was designed by introducing the finite element model into the self-designed model based on the structural characteristics of the cerebral blood vessels and the commercial Solitaire stent.

### 2.2 Design of thrombus removal devices

The commercial Solitaire stent was used as a reference and imported using a finite element model [7,8]. In brief, an unfolded 3D model of the stent was established using SOLIDWORKS. The model was imported into Abaqus, where it was reasonably sliced to capture the local peak stresses and strains in the bracket. The stent was discretized into an eight-node hexahedral reduced integral cell (C3D8R) model. By mesh convergence analysis, a four-layer mesh was set up in the thickness direction of the bracket. It was then exported as an *inp file and the node coordinates in the *inp file were then replaced with the coordinate information after MATLAB coordinate transformation. Finally, the replaced *inp file was imported into Abaqus to complete the import of the whole model. Through the above finite element simulation method, stent modeling, *in vitro* bending simulation, stent maximum deformation, radial force simulation, and in-vessel unfolding simulation were carried out. The results of thrombotic stent removal were evaluated for different sizes and friction characteristics.

The radial forces of the Solitaire stent and the gradual diameter stent were analyzed using finite element analysis at 4.38 and 7.07 N. This led to the conclusion that the gradual diameter stent has better support for the vessel wall when deployed. Ultimately, we designed an open-ended variable-diameter stent structure containing different variations in rib width and wall thickness dimensions. The Abaqus stent was compared with a commercial Solitaire 4 × 40 stent in terms of flexibility, radial force, and interaction with the vessel wall, and the stent structure was selected for superior performance. The selection criteria included a smaller difference in diameter size to vessel diameter, more adequate deployment after implantation in the distal vessel, anatomical compliance with the intracranial vasculature and adaptation to the diseased arterial vessel, and good longitudinal flexibility, i.e., the bending characteristics of the stent. This makes it easier to cross and reach the diseased vessel, reducing damage to the vessel wall and minimizing the chance of vascular injury while providing efficient embolization. This makes embolization procedures more efficient and safe and provides theoretical support for the development and application of new embolization devices.

### 2.3 Stent model

**Figure 1:**
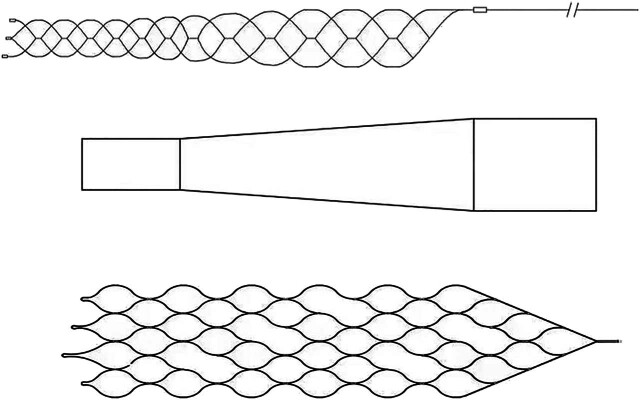
Stent model and unfolding model.

The main part of the stent consisted of eight axially extending sinusoidal bars. The sinusoidal bars were staggered circumferentially to form a porous tubular structure of the holder. The proximal end of the bracket was conical and connected to the push rod to facilitate the recovery and release of the stent. The connecting segments were evenly removed from the stent axis to improve the smoothness of the stent. The designed stent was structurally asymmetrical and was prepared using laser-cut nickel-titanium alloy tubes. The material nitinol, a shape memory alloy, has excellent properties such as corrosion resistance, super-elasticity, high damping, and biocompatibility. It was widely used in the medical field and has become the material of choice for self-expanding stents. The stent is shown in Figure 1. We performed subsequent experimental validation of the preferred stent.

### 2.4 Preparation of thrombus

**Figure 2:**
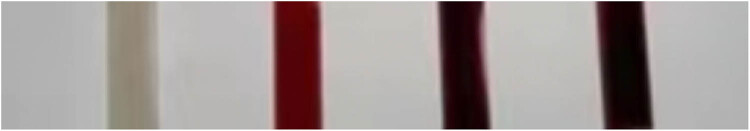
Four thrombi with 0, 5, 40, and 80% RBC volume content.

The Chandler Loop technique was a common technique for creating dynamic thrombus [9–11]. This technique was used with respective devices to generate different types of thrombus. Citrated whole blood was obtained from sheep venous blood and collected after quality control. Materials included sheep venous blood with 3.2% sodium citrate (1:9) anticoagulated, 2.06% calcium chloride (1:9) procoagulant, and centrifuged in a 37°C water bath. (1) Take the sodium citrate anticoagulated blood and centrifuge at 550×*g* for 15 min to separate the whole blood into three different layers of plasma, white blood cells, and RBC. (2) Take different proportions of plasma and RBC volume ratio and mix well, add 2.06% calcium chloride at 1:9, and react for 30 min in a 37°C water bath. (3) Produce four thrombi with 0, 5, 40, and 80% RBC volume content, as shown in Figure 2. The thrombus with 0% RBC volume is rich in fibrin.

### 2.5 *In vitro* cerebrovascular circulation model

**Figure 3:**
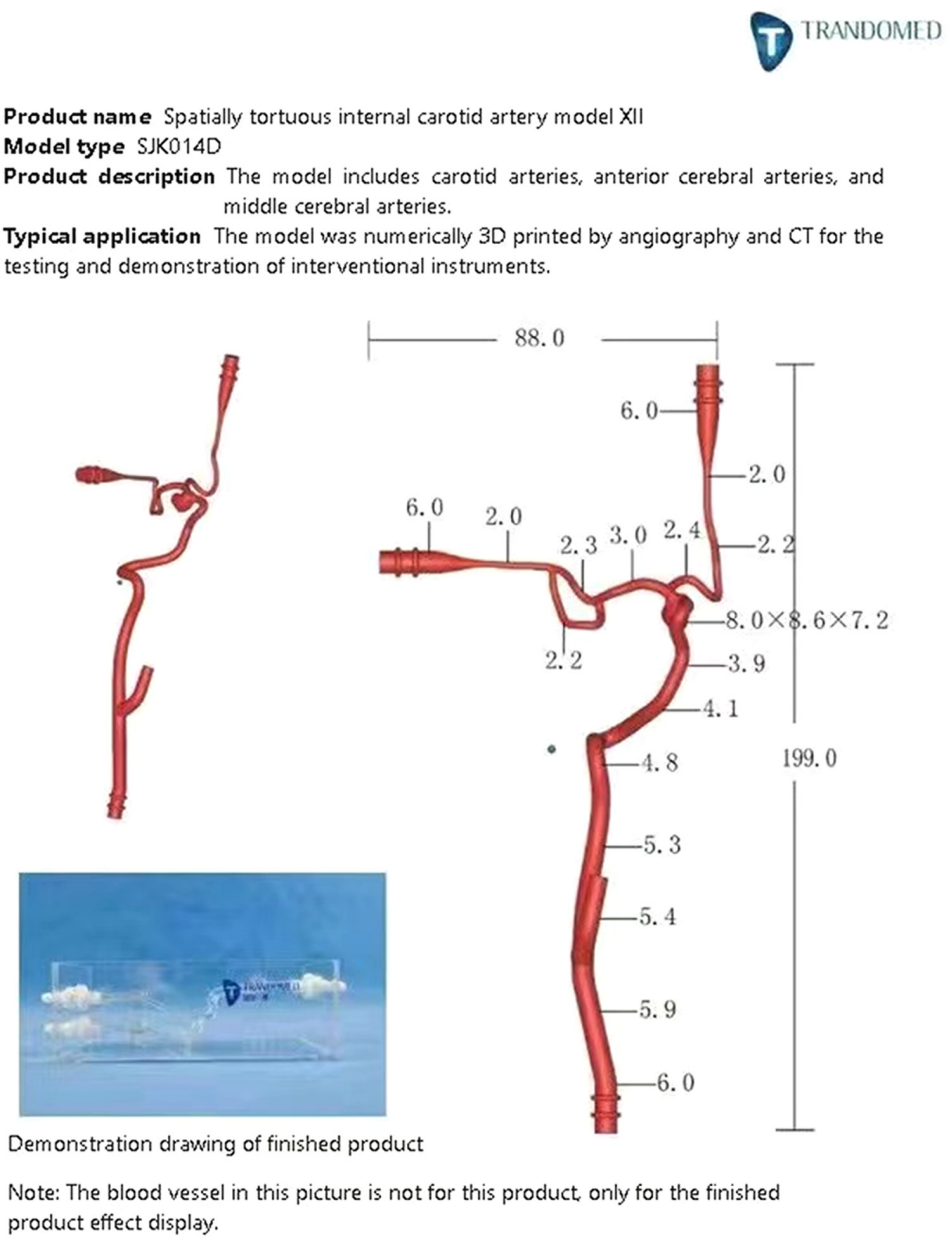
Common carotid tortuosity.

A silicone vascular model was prepared based on the soft model described by Mashiko et al. [12]. Vascular data of different tortuosity grades from whole brain angiography (DSA) images were imported into the Vitrea 3D workstation for data modeling. After a series of processing, the final stereolithography file was exported. A model of the common carotid artery tortuosity was finally printed in silicone on a 3D printer (Figure 3). Each internal carotid artery (ICA) comprised the anterior cerebral artery and the middle cerebral artery (MCA), including the M2 zone. Their vascular models were then integrated into a circulatory system, including a 37°C saline reservoir and a miniature water pump. We used 3D printing technology to simulate the anatomical features of real blood vessels as closely as possible.

### 2.6 Stenting procedure

The fabricated silicone *in vitro* carotid cerebrovascular circulation model was fixed on the test table. It was circulated by injecting a 0.9% sodium chloride solution at 37°C into it via a mini temperature-controlled pump. The thrombus product was placed at the target location of the carotid artery model as the site of vascular occlusion, and then an arterial catheter sheath was placed in the ICA. A black loach guidewire was used to guide the Navien catheter to the cavernous sinus segment and a micro-guide to guide the microcatheter to the distal end of the thrombus. After securing the Y-valve, the stent was pushed through the microcatheter until it exceeded the thrombus, ensuring that the thrombus was located in the mid-posterior portion of the effective stent length. The stent was released by slowly retracting the microcatheter in a proximal direction and held in position for 5 min after release. The stent and microcatheter were then back-spread as a whole until the stent was spilled to observe the effect of thrombus removal. Using this model, the two stent systems were tested and compared for embolization efficiency.

### 2.7 Grouping and thrombus removal procedure

The thrombus products were selected for preparation. One experimental group and one control group were set up for each type of artificial thrombus. One commercial thrombus retrieval stent, the SOLITAIRE stent 6 × 30 mm (Medtronic, California, USA), and the presently developed gradual diameter thrombectomy stent were used for comparison of thrombus removal. Each group contained four thrombotic products with RBC volume contents of 0, 5, 40, and 80%, respectively.

Thrombi prepared with 0% RBC volume content (approximately 10 mm in length) were placed into the M1 segment of the circulatory system of the vascular model to simulate an occluded vessel. The cerebral vascular model was then connected to a 37°C saline reservoir and a micro-pump (collectively referred to as a mini temperature-controlled pump). A peristaltic pump was used to circulate through the model to ensure physiologically accurate hemodynamics. The thrombus was allowed to remain in the target location of the model vessel for at least 10 min. At the end of the retention time, the microcatheter was guided through the Navien catheter with a micro-guide wire to the distal end of the thrombus. The Solitaire clot retrieval stent and introducer sheath were placed in the anterior segment of the microcatheter until the anterior end of the sheath was confirmed in place. The Y-valve was secured and the stent was pushed into the microcatheter. Once the soft part of the pushing wire was fully inserted into the microcatheter, the introducer sheath was removed after 10 cm of advancement. Push the clot retrieval stent until it passes the thrombus, ensuring that the thrombus is at the mid-posterior part of the effective length of the stent. Secure the pushing guidewire to keep the stent in place. At the same time, the microcatheter was slowly retracted in a proximal direction to release the stent and held in place for 5 min after release. The stent and microcatheter were then spread back as a whole until the retrieval stent was spread out and the retrieval was complete, and the removal was observed. During this procedure all thrombi were satisfactorily removed in the least number of removals possible, allowing recanalization of the occluded simulated vessel. In the same way, the gradual diameter stent developed here was placed through a microcatheter at the site of the thrombus with 0% RBC volume. The same procedure as above was followed to remove the thrombus and to observe the effect of thrombus removal. The control and experiment stents were used five times for comparison.

The procedure was repeated with prepared thrombi at 5, 40, and 80% RBC volume, respectively. Five experiments were performed using this device for each type of thrombus, 20 experiments for each device, for a total of 40 experiments.

### 2.8 Observed indicators

The first-pass recanalization rate (FPR) is defined as the FPR achieved after the first thrombolysis [6]. If the occlusion is still present after the first embolization, the one-time recanalization rate is negative, and up to two embolizations can be performed to reopen the vessel. All two embolization methods were counted in one experiment. Second, the degree of thrombus and stent embedding was observed during each experiment. The displacement of the thrombus in the stent was observed and collected at the end of the experiment.

### 2.9 Statistical analysis

The FPR for each type of thrombus taken with each stent was recorded and observed separately. Raw values were recorded for the count data. The Kruskal–Wallis test, chi-square (*χ* ^2^) test, and two-way ANOVA were used to analyze the effect of thrombus and stent type on the outcome of thrombus extraction. The statistical significance level *α* = 0.05. The Bonferroni method was used for the correction of multiple comparisons. *p* < 0.05 was significantly different.

## 3 Results

### 3.1 Comparison of recanalization and thrombus displacement

Different types of thrombi were taken in an *in vitr*o cerebrovascular model using a gradual diameter thrombectomy stent (experimental group) and a Solitaire stent (control group). The recanalization (Table 1) and thrombus displacement (Table 2) were observed for each thrombus when the same experiment was repeated five times. No specific data were presented as no relevant indicators or literature on thrombus-stent embedding were available and the degree of embedding could only be observed visually during the removal of the thrombus. It was shown that the recanalization was 100% for both groups for the *in vitro* thrombus removal experiments in this location.

**Table 1:** Comparison of the recanalization rate after the first thrombus removal between the experimental and control groups

Groups	Items	0% RBC volume	5% RBC volume	40% RBC volume	80% RBC volume
Gradual diameter thrombectomy stent	Time	5	5	5	5
	Success rate (%)	100	100	100	100
Solitaire stent	Time	5	5	5	5
	Success rate (%)	100	100	100	100

**Table 2:** Comparison of the thrombus displacement between the experimental and control groups

Groups	Items	0% RBC volume	5% RBC volume	40% RBC volume	80% RBC volume
Gradual diameter thrombectomy stent	Time	5	5	5	5
	Displacement	Severe	Moderate	Mild	Mild
Solitaire stent	Time	5	5	5	5
	Displacement	Severe	Moderate	Mild	Mild

A thrombus displaced less than half the length of the thrombus itself is considered to be mild translocation, less than half the length of the thrombus itself and greater than half the length of the thrombus is considered to be moderate translocation, and greater than the thrombus itself is considered to be severe translocation.

### 3.2 Comparison of the embedding degree of thrombus and stent

The embedding degree of thrombus and stent was observed during each experiment (Table 3). Whether the thrombus was taken from 0 to 80% of the RBC volume with the gradual diameter thrombectomy stent or with the Solitaire stent, the thrombus containing 0% of the RBC volume (rich in fibrin) was poorly embedded with the stent, while the stent was relatively poorly embedded with the thrombus in the more tortuous part of the vessel and the stent did not open well in this part of the vessel.

**Table 3:** Comparison of the embedding degree of thrombus and stent between the experimental and control groups

Groups	Items	0% RBC volume	5% RBC volume	40% RBC volume	80% RBC volume
Gradual diameter thrombectomy stent	Time	5	5	5	5
	Embedding degree	Poor	General	Good	Good
Solitaire stent	Time	5	5	5	5
	Embedding degree	Poor	General	General	General

Thrombosis of over 80% embedded in the stent is considered good, 50–80% is considered general, and less than 50% is considered poor.

### 3.3 Comparison of thrombus removal capacity

**Figure 4:**
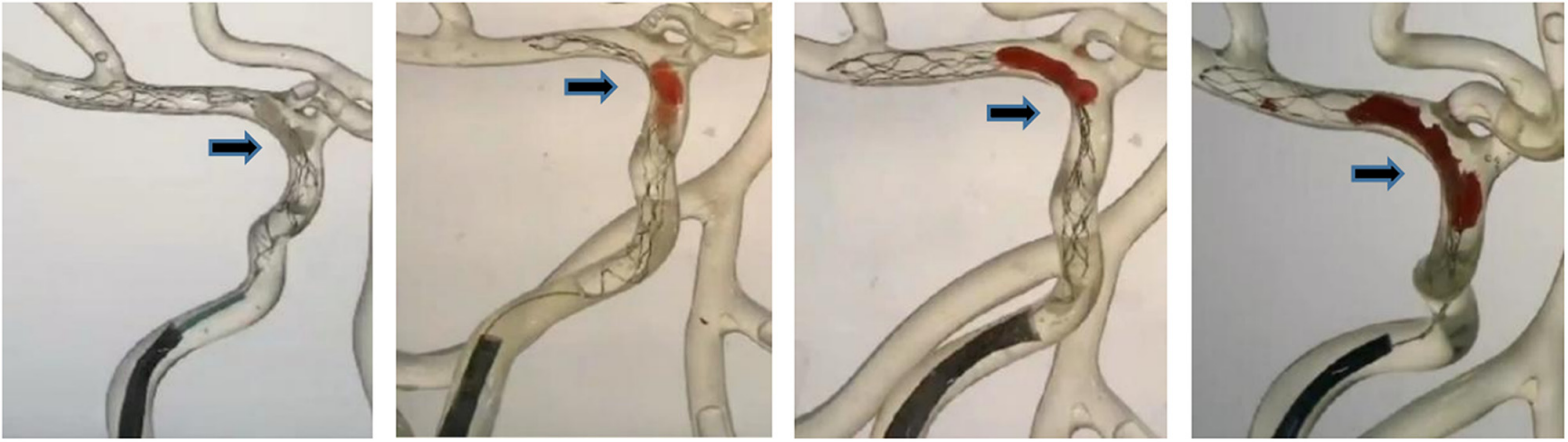
State of the gradual diameter thrombectomy stent in a tortuous area. Thrombus containing 0, 5, 40, and 80% of the RBC volume from left to right, with arrows indicating the opening of the tapering diameter stent at the tortuous site of the vessel.

**Figure 5:**
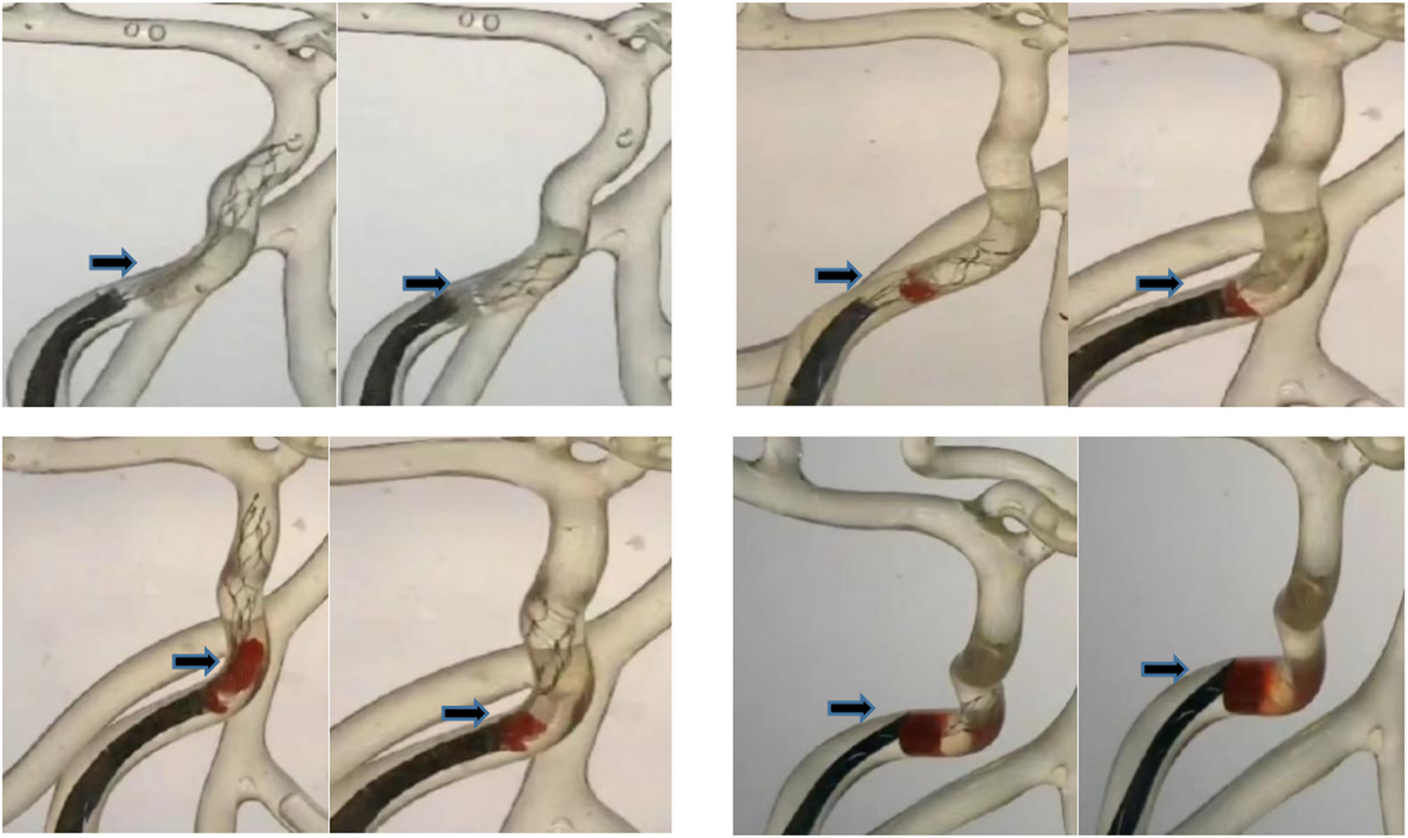
Different thrombi containing 0–80% of the RBC volume migrate to varying degrees distal to the stent. Thrombus containing 0, 5, 40, and 80% of the RBC volume from left to right, with arrows indicating migration of blood clots at the distal end of a graduated diameter stent.

Figure 4 shows the state of the gradual diameter thrombectomy stent in a tortuous area. When the gradual diameter thrombectomy stent or the Solitaire stent was incorporated into the introducer catheter along with the delivery catheter, different thrombi containing 0–80% of the RBC volume migrated to varying degrees distal to the stent (Figure 5). As the RBC volume contained increased, more thrombi fragmented and escaped, most notably with thrombi containing 80% of RBC volume. This showed that there was no effect of change in thrombus type and stent type on the outcome of thrombus removal. There was no significant difference in the thrombus removal capacity of the graduated diameter thrombectomy stent and the Solitaire stent. The possibility that the experimental thrombus removal setup was too simple cannot be ruled out.

## 4 Discussion

In this *in vitro* experiment, acute thrombotic occlusion of the MCA was mimicked by four different types of artificial thrombi. We evaluated the effectiveness of the gradual diameter thrombectomy stent, measured the recanalization rate after the first retrieval, and observed the degree of embedding of the different thrombi with the stent and distal migration of different thrombi during stent retrieval to the introducer catheter. The results of other types of thrombus retrieval stents in vascular models have been reported with different results [13–15]. However, comparisons with these studies should be made with caution due to the wide variation in experimental design. Nevertheless, in the current study, the gradual diameter thrombectomy stent for thrombectomy demonstrated a high primary recanalization rate. Intracranial vessels are thinner and more curved, which may have a significant impact on the flexibility, radial force, and performance of the embolization stent [15,16]. In addition, a balloon-guided catheter was not used in our experiments to block blood flow proximally. This is a system commonly used in intracranial embolization and can be effective in reducing embolism [17,18]. In the case of smaller cerebral vessel diameters, it can also be assumed that the diameter of the aspiration catheter itself has caused some occlusion of the vessel lumen, thus reducing the risk of embolization.

Our experiments also provide further evidence of the importance of thrombus structure. The smaller the erythrocyte-containing volume, the more stable it is, and the larger the thrombus, the more fragile it is and the higher the propensity for fragmentation and embolization. However, thrombi containing a small volume of RBC are rich in fibrin and are less well embedded in the stent, making it more difficult to remove the stent. Many clinical and *in vitr*o studies have demonstrated the high friction and low success rate of thrombus retrieval in fibrin-rich thrombi [19–21]. The nature of the thrombus, as well as the length and severity of the tortuosity of the vessel, makes thrombus removal by stent retrievers challenging.

This study describes the dynamic process of stent retrieval with a gradual diameter thrombectomy stent and its interaction with different types of thrombus. The main results are that the gradual diameter thrombectomy stent is as effective as currently used stents *in vitro* experiments, with a high primary recanalization rate, but also with poor stent-thrombus embedding, especially in the more tortuous parts of the vessel. This is related to the severe deformation of the stent here and the increased friction between the stent and the vessel because it is an experimental rather than a commercial device, and there is much room for improvement. Due to the mechanical properties of the stent itself, it can also cause thrombus migration distal to the stent. The entity of the thrombus and its associated components and mechanical properties have an impact on the effectiveness and safety of the device. In particular, thrombi with a high erythrocyte content are more fragile. In contrast, thrombi with a lower erythrocyte content (rich in fibrin) are tougher. As a result, a higher risk of embolism and lower effectiveness are associated. When the embolization device is withdrawn into the recovery catheter together with the delivery microcatheter, giving negative pressure to the suction catheter, i.e., suction through the intermediate catheter technique (SAVE technique) has a positive effect on preventing distal migration of the thrombus in the stent and can better improve the effectiveness of the embolization [22].

There are some limitations to this experiment. A thrombus product was used to test stent removal. Although the pressure conditions within the model mimic the physiological conditions of blood circulation and the prepared thrombi are somewhat heterogeneous, the study lacks certain *in vivo* characteristics, including atherosclerotic vessel walls, hemodynamic changes during the intervention, and thrombus–vessel wall interactions. In future study, we will further explore its characteristics *in vivo* model.

## 5 Conclusion

The present experimental study confirms the good recanalization rate of the gradual diameter thrombectomy stent compared to the conventional stent, and the high effect of thrombus removal. However, further experimental and clinical studies are needed on its safety and efficacy.
